# Association between red blood cell distribution width-to-albumin ratio and all-cause mortality in intracerebral hemorrhage

**DOI:** 10.3389/fnut.2025.1599104

**Published:** 2025-06-04

**Authors:** Jia Xu, Guangdong Wang, Xinran Chen, Xinyi Xu, Yun Wang, Li Wang, Yaxin Zhang

**Affiliations:** ^1^Department of Electrocardiography Diagnosis, The Second Affiliated Hospital of Anhui Medical University, Hefei, Anhui, China; ^2^Department of Respiratory and Critical Care Medicine, First Affiliated Hospital of Xi’an Jiaotong University, Xi’an, Shanxi, China; ^3^Department of Neurology, Xiamen Humanity Hospital, Fujian Medical University, Xiamen, Fujian, China

**Keywords:** red blood cell distribution width-to-albumin ratio, intracerebral hemorrhage, mortality risk, prognosis, risk factor

## Abstract

**Background:**

Intracerebral hemorrhage (ICH) remains a devastating cerebrovascular condition, marked by high fatality and limited availability of prognostic tools. The red blood cell distribution width-to-albumin ratio (RAR) has recently gained attention as a composite biomarker of systemic inflammation and nutritional condition, but its prognostic value in ICH remains unclear. We aim to examine how RAR relates to mortality risk among individuals with ICH.

**Methods:**

We performed a retrospective cohort analysis using the Medical Information Mart for Intensive Care-IV database. A total of 1,410 ICH individuals hospitalized in the intensive care unit were included and categorized into quartiles according to their RAR levels. The primary endpoint was all-cause mortality at 365 days, while 90-day all-cause mortality served as a secondary endpoint. Kaplan–Meier survival analysis, time-varying Cox regression model, and restricted cubic spline analyses (RCS)analysis were performed to assess the link between RAR and mortality risk. The predictive utility of RAR was further assessed through receiver operating characteristic (ROC)curve. Subgroup analyses explored potential effect modifications.

**Results:**

Among the 1,410 ICH patients analyzed, the median age was 69 years. The all-cause mortality rates at 90-day and 365-day were 35.53 and 42.62%, respectively. Individuals with the highest RAR levels experienced significantly greater 90 days (54.34% vs. 21.97%, *p* < 0.001) and 365 days (62.18% vs. 29.77%, *p* < 0.001) than those with the lowest levels. Time-varying Cox regression model revealed that increased RAR levels were significantly and independently linked to greater mortality risk (hazard ratios [HR] for 365-day mortality:1.07, 95% CI:1.02–1.13, *p* = 0.005; HR for 90-day mortality: 1.14, 95%CI: 1.05–1.12, *p* = 0.001). ROC curve analysis demonstrated that combining RAR with the SOFA score improved predictive accuracy for 90-day and 365-day. RCS analyses indicated a nonlinear connection between higher RAR values and mortality rates. Subgroup analyses revealed that a largely uniform effect of RAR across different subpopulations except for age, gender, and race.

**Conclusion:**

An elevated RAR is independently and significantly associated with increased all-cause mortality in ICH patients, regardless of established risk predictors. Its combination with the SOFA score enhances prognostic accuracy. These results suggest its potential clinical utility for early risk stratification.

## Introduction

Intracerebral hemorrhage (ICH) continues to pose a critical clinical burden owing to its considerable global impact on functional impairment and fatality (1). Accounting for approximately 10–20% of all strokes, ICH carries a significantly worse prognosis than ischemic stroke (2, 3). Despite advancements in diagnostic techniques and therapeutic strategies, ICH remains a major clinical challenge, often resulting in severe neurological deficits, substantial healthcare costs, and considerable burden to patients and their families (4). Given these challenges, the identification of accessible and reliable biomarkers for early prognostic assessment in ICH is essential for improving clinical outcomes.

The red blood cell distribution width-to-albumin ratio (RAR) is a novel hematological parameter reflecting both inflammation and nutritional status. Red blood cell distribution width (RDW) functions as a recognized marker of variability in erythrocyte size and systemic inflammation (5), while serum albumin reflects both nutritional condition and overall systemic homeostasis. Integrating these two parameters, RAR has recently gained attention as a potential prognostic biomarker in various disease states, including acute pancreatitis and rheumatoid arthritis (6–9). As inflammation and nutritional condition are both key contributors to the underlying mechanisms of ICH (10, 11), RAR may hold prognostic value for risk assessment in this group of patients. However, its clinical relevance and prognostic utility in ICH remain unclear, necessitating further investigation.

The study explores the link between RAR and outcomes in ICH patients using data extracted from the Medical Information Mart for Intensive Care (MIMIC)-IV database. Specifically, we seek to determine whether RAR can serve as a readily accessible and cost-effective biomarker to identify ICH patients likely to experience unfavorable clinical outcomes. The findings of this study may contribute to improved risk stratification, facilitate early interventions, and ultimately lead to better clinical decision-making and recovery prospects for ICH patients.

## Materials and methods

### Data source

The MIMIC-IV database (version 3.1) is a publicly available, de-identified critical care database that integrates comprehensive electronic health records (EHRs) of patients admitted to the intensive care units (ICUs) at Beth Israel Deaconess Medical Center (BIDMC), Boston, Massachusetts. Developed by the Laboratory for Computational Physiology at the Massachusetts Institute of Technology (MIT), MIMIC-IV serves as a critical resource for epidemiological research, predictive modeling, and machine learning applications in critical care medicine. By enabling large-scale retrospective studies, it significantly contributes to advancements in clinical research and patient care. The first author of this study complied with all ethical and regulatory requirements for accessing MIMIC-IV, including completion of the necessary research ethics training and obtaining appropriate credentials (Certification ID: 64822128). The first author is also responsible for data extraction and preprocessing, ensuring adherence to the database’s guidelines and ethical considerations.

### Study population, data extraction, and clinical outcomes

**Figure 1:**
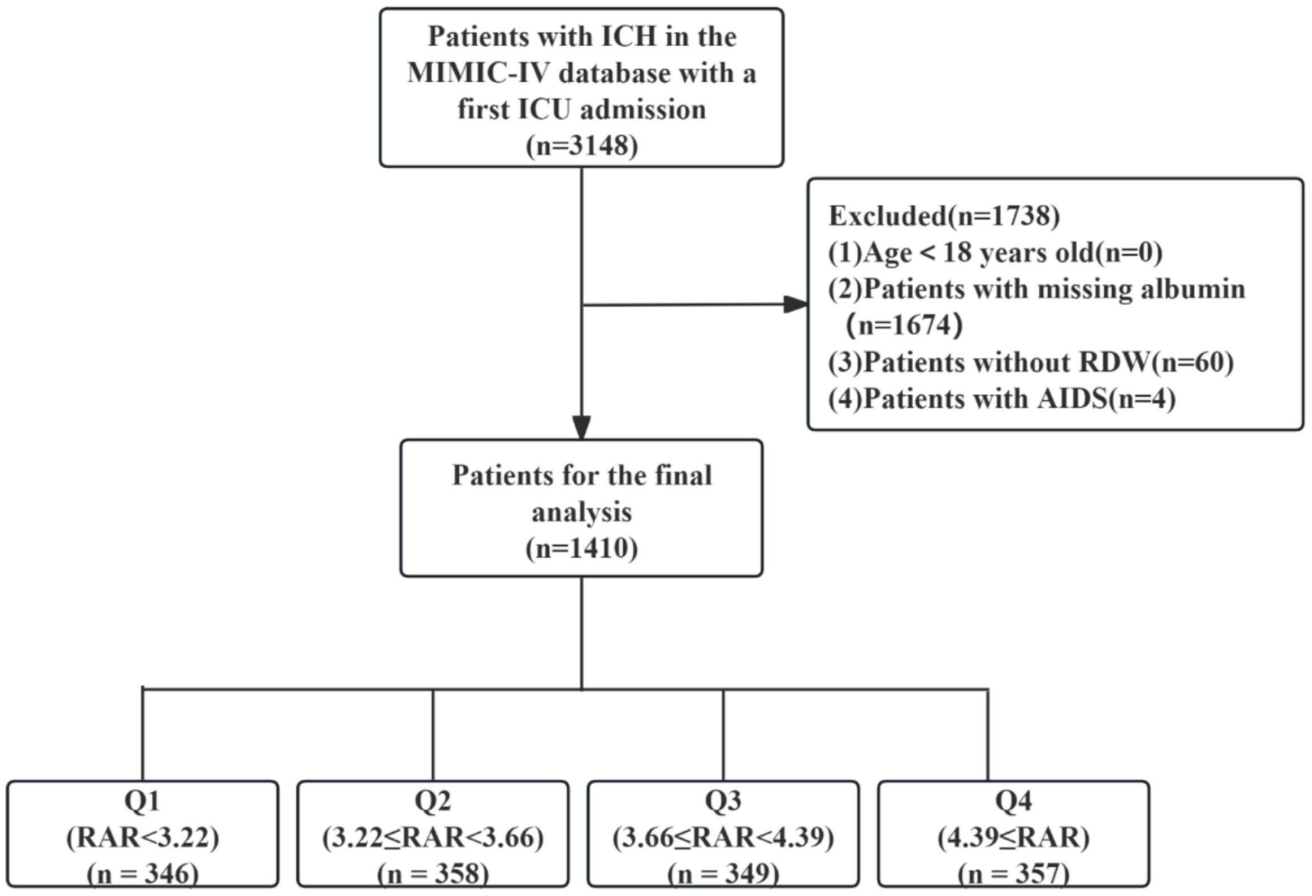
Flow chart of population selection. ICH, intracerebral hemorrhage; MIMIC, Medical Information Mart for Intensive Care; ICU, intensive care unit; RDW, red cell distribution width; AIDS, acquired immune deficiency syndrome; RAR, red blood cell distribution width to albumin ratio.

We identified 3,148 ICH patients with initial ICU admissions, confirmed through ICD-9 and ICD-10 diagnostic coding. Among them, only adult individuals (≥18 years old) were considered. Participants were excluded if they had missing key laboratory data necessary for calculating RAR, including RDW and serum albumin post ICU admission. Additionally, patients with acquired immunodeficiency syndrome were omitted. After applying the selection standards, 1,410 participants entered the study and proceeded to the next phase of assessment. Patients were then stratified into quartiles based on their RAR levels for comparative analysis (Figure 1).

In our investigation, clinical information was retrieved from the MIMIC-IV database via PostgreSQL software. To ensure consistency, only parameters documented during the initial 24-h period following ICU entry were included. The first recorded result within this period was considered for each laboratory parameter. Extracted variables encompassed five key domains: demographic information, clinical severity scores, vital signs, comorbidities, laboratory test results, and treatment measures. A detailed summary of these variables is provided in Table 1. To minimize potential bias, features exhibiting over 10% missing values were removed. Regarding the remaining missing data (Supplementary Table S1), multiple imputation was conducted using a random forest-based approach via the “mice” package in R.

**Table 1:** The detailed extracted variables in MIMIC-IV database.

Items	Composition
Demographic information	Age; Gender; Race
Clinical severity scores	SOFA; GCS; LODS
Vital signs	SBP; DBP; Heart rate; Respiratory rate; Temperature; SpO2
Comorbidities	Hypertension; Diabetes; Myocardial infarction; Congestive heart failure; Peripheral vascular disease; Chronic pulmonary disease; AKI; Sepsis
Laboratory test results	WBC; RDW; Albumin; Platelet; BUN; Creatinine; Potassium; Sodium; Glucose; INR; Anion gap; PT
Treatment measures	Vasoactive drug; Statin; Mannitol; Beta blockers; Diuretic; Ventilator; CRRT; Cerebral surgery

SOFA, sequential organ failure assessment; GCS, Glasgow coma scale; LODS, Logistic Organ Dysfunction System; SBP, Systolic Blood Pressure; DBP, Diastolic Blood Pressure; SpO2, oxygen saturation; AKI, acute kidney injury; WBC, white blood cell; RDW, red cell distribution width; BUN, blood urea nitrogen; INR, international normalized ratio; PT, prothrombin time; CRRT, continuous renal replacement therapy.

The value of the RAR was obtained as [RDW (%)/serum albumin (g/dL)]. These data were systematically processed for subsequent statistical analysis.

The primary endpoint of the study was all-cause mortality within 365 days of hospital admission, while the secondary endpoint was all-cause mortality within 90 days, both defined as death occurring within the respective timeframes post-admission.

### Statistical analysis

Normality of continuous variables was evaluated using the Kolmogorov–Smirnov test. As none followed a normal distribution, data were represented as medians with interquartile ranges (IQRs), and the Mann–Whitney U test was applied to evaluate differences across groups. Categorical variables were summarized as percentages (%), with differences between groups assessed via Pearson’s chi-squared test. Kaplan–Meier analyses were applied to assess cumulative survival across RAR quartiles. To identify mortality risk factors, the least absolute shrinkage and selection operator (LASSO) regression was applied, using L1 regularization to refine variable selection by shrinking coefficients and discarding non-contributory variables. The optimal value of the tuning parameter (*λ*) was determined through 10-fold cross-validation. This method allows us to iteratively evaluate the model’s performance on different subsets of the data, and select the λ that minimizes the prediction error while avoiding overfitting. Given the potential correlation of variables, we assessed multicollinearity by calculating the Variance Inflation Factor (VIF) for each variable. All the VIF values were found to be less than 5, indicating that multicollinearity is not a significant issue in our model (Supplementary Table S3). Proportional hazards assumptions were assessed using Schoenfeld residuals. In cases where violations were detected, time-dependent effects were incorporated into the Cox model to account for changes over time (Supplementary Table S4). Multivariate Cox model by RAR as a time dependent covariate were developed to verify how RAR correlates with clinical outcomes, incorporating adjustments in stepwise models. Model 1 was a crude model, while Model 2 included age, gender, and race. Model 3 accounted for additional covariates, such as Sequential Organ Failure Assessment (SOFA), sepsis, blood urea nitrogen (BUN), potassium, glucose, Logistic Organ Dysfunction System (LODS), vasoactive drugs, statins, mannitol, continuous renal replacement therapy (CRRT), cerebral surgery, and length of hospital stay. RAR was analyzed as both a continuous and categorical variable, with the lowest quartile serving as the reference category. A trend test across quartiles was conducted to assess the significance of RAR’s association with mortality risk. To evaluate the RAR’s ability to distinguish outcomes, receiver operating characteristic (ROC) curve was conducted. DeLong’s test was used to confirm whether the observed increase in the area under the curve (AUC) is statistically significant. Decision curve analysis (DCA) was conducted to assess the added clinical value of the models. To evaluate the incremental predictive ability of RAR over RDW or albumin alone, we constructed fully adjusted models (Model 3) using each marker individually. We compared the models’ discriminative performance using the AUC via DeLong’s test. Additionally, Net Reclassification Improvement (NRI) and Integrated Discrimination Improvement (IDI) were calculated to assess the degree of improvement in classification and discrimination. Nonlinear relationships of RAR with mortality outcomes were evaluated through restricted cubic spline (RCS) regression with four knots. Additionally, RAR was dichotomized at the median and included in stratified analyses across subgroups, including age, gender, race, hypertension, diabetes, acute kidney injury (AKI), myocardial infarction (MI), chronic pulmonary disease (COPD), peripheral vascular disease (PVD), sepsis, and Geriatric Nutritional Risk Index (GNRI). Interaction modifications involving RAR and subgroup characteristics were tested via likelihood ratio testing. All statistical analyses were two-tailed, with a significance threshold of *p* < 0.05. Statistical procedures were conducted with SPSS and R.

## Results

This research included 1,410 ICH cases, with participants having a median age of 69 years (IQR: 58–80) (Table 2). Of these, 791 (56.10%) were male and 826 (58.78%) were White. The median hospital stay was 8.57 days (IQR: 4.18–16.13), while the median ICU stay was 3.92 days (IQR: 1.88–8.62). At 90 and 365 days, the all-cause mortality rates reached 35.53 and 42.62%, respectively.

**Table 2:** Characteristics and clinical outcomes of participants categorized by RAR.

Characteristics	Total (*n* = 1,410)	Q1 (RAR < 3.22) (*n* = 346)	Q2 (3.22 ≤ RAR < 3.66) (*n* = 358)	Q3 (3.66 ≤ RAR < 4.39) (*n* = 349)	Q4 (4.39 ≤ RAR) (*n* = 357)	*p*-value
Age (year)	69.00 (58.00, 80.00)	67.00 (55.00, 77.00)	70.00 (61.00, 81.00)	73.00 (62.00, 82.00)	68.00 (58.00, 80.00)	<0.001
Gender, *n* (%)						0.180
Female	619 (43.90)	136 (39.31)	155 (43.30)	162 (46.42)	166 (46.50)	
Male	791 (56.10)	210 (60.69)	203 (56.70)	187 (53.58)	191 (53.50)	
Race, *n* (%)						0.706
Non-White	584 (41.42)	139 (40.17)	144 (40.22)	144 (41.26)	157 (43.98)	
White	826 (58.58)	207 (59.83)	214 (59.78)	205 (58.74)	200 (56.02)	
Vital signs						
Heart rate (beats/min)	82 (71, 94)	81.00 (70, 92)	80.50 (70, 92)	81.00 (72, 93)	86.00 (73, 101)	<0.001
SBP (mmHg)	137 (122, 151)	138 (124, 150)	138 (123, 152)	138 (124, 153)	132 (114, 146)	<0.001
DBP (mmHg)	76 (65, 88)	78 (67, 91)	78 (65, 88)	77 (65, 89)	71 (61, 85)	<0.001
Respiratory rate (times/min)	18 (16, 22)	18 (16, 21)	18 (15, 22)	18 (16, 21)	19 (16, 23)	0.025
Temperature (°C)	36.8 (36.5, 37.1)	36.8 (36.6, 37.1)	36.9 (36.6, 37.1)	36.8 (36.4, 37.2)	36.8 (36.5, 37.2)	0.375
SpO2 (%)	98 (96, 100)	98 (96, 100)	98 (96, 100)	98 (96, 100)	99 (96, 100)	0.594
Severity scores						
SOFA	3 (2, 5)	2 (1, 4)	3 (2, 4)	3 (2, 5)	5 (3, 7)	<0.001
GCS	14 (11, 15)	14 (12, 15)	14 (11, 15)	14 (11, 15)	14 (11, 15)	0.608
LODS	3 (2, 5)	3 (2, 4)	3 (2, 5)	4 (2, 5)	5 (3, 7)	<0.001
Comorbidity, *n* (%)						
Hypertension	1,135 (80.50)	283 (81.79)	300 (83.80)	295 (84.53)	257 (71.99)	<0.001
Diabetes	372 (26.38)	65 (18.79)	84 (23.46)	114 (32.66)	109 (30.53)	<0.001
Myocardial infarction	147 (10.43)	19 (5.49)	34 (9.50)	36 (10.32)	58 (16.25)	<0.001
Congestive heart failure	229 (16.24)	22 (6.36)	49 (13.69)	69 (19.77)	89 (24.93)	<0.001
Peripheral vascular disease	77 (5.46)	6 (1.73)	25 (6.98)	26 (7.45)	20 (5.60)	0.003
Chronic pulmonary disease	27 (7.80)	32 (8.94)	57 (16.33)	60 (16.81)	27 (7.80)	<0.001
AKI	1,083 (76.81)	242 (69.94)	268 (74.86)	272 (77.94)	301 (84.31)	<0.001
Sepsis	614 (43.55)	105 (30.35)	126 (35.20)	162 (46.42)	221 (61.90)	<0.001
Laboratory parameters						
WBC (K/μL)	10.1 (7.8, 13.5)	10.4 (8.0, 13.1)	9.8 (7.8, 12.8)	9.9 (8.0, 13.8)	10.2 (7.5, 14.4)	0.630
Platelet (K/μL)	200.0 (155.0, 256.0)	209.5 (170.0, 248.8)	206.0 (169.0, 252.3)	198.0 (151.0, 256.0)	180.0 (117.0, 269.0)	<0.001
BUN (mg/dL)	17.0 (12.0, 23.0)	14.0 (11.0, 18.0)	16.0 (12.0, 21.0)	17.0 (14.0, 26.0)	20.0 (13.0, 33.0)	<0.001
Creatinine (mg/dL)	0.9 (0.7, 1.2)	0.9 (0.7, 1.0)	0.9 (0.7, 1.1)	1.0 (0.8, 1.3)	1.0 (0.7, 1.5)	<0.001
Potassium (mmol/L)	4.0 (3.6, 4.3)	3.9 (3.6, 4.2)	4.0 (3.7, 4.3)	4.0 (3.7, 4.4)	4.0 (3.6, 4.4)	0.032
Sodium (mmol/L)	139.0 (137.0, 142.0)	140.0 (137.0, 142.0)	140.0 (137.0, 142.0)	140.0 (137.0, 142.0)	139.0 (135.0, 142.0)	0.007
Glucose (g/dL)	130.0 (107.0, 161.0)	131.5 (109.0, 158.0)	126.0 (104.0, 153.0)	133.0 (109.0, 167.0)	130.00 (104.0, 169.0)	0.150
INR	1.2 (1.1, 1.3)	1.1 (1.0, 1.2)	1.1 (1.1, 1.2)	1.2 (1.1, 1.3)	1.3 (1.1, 1.6)	<0.001
Anion gap (mmol/L)	15.0 (13.0, 17.0)	15.0 (13.0, 17.0)	14.0 (13.0, 16.0)	15.0 (13.0, 17.0)	15.0 (12.0, 17.0)	<0.001
PT(s)	12.7 (11.7, 14.4)	12.0 (11.3, 13.0)	12.5 (11.7, 13.7)	12.9 (11.9, 14.4)	14.2 (12.6, 17.0)	<0.001
Treatment, *n* (%)						
Beta blockers	580 (41.13)	137 (39.60)	143 (39.94)	153 (43.84)	147 (41.18)	0.659
Diuretic	486 (34.47)	105 (30.35)	101 (28.21)	119 (34.10)	161 (45.10)	<0.001
Mannitol	187 (13.26)	56 (16.18)	49 (13.69)	39 (11.17)	43 (12.04)	0.221
Statin	446 (31.63)	100 (28.90)	129 (36.03)	97 (27.79)	120 (33.61)	0.060
Vasoactive drug	263 (18.65)	43 (12.43)	48 (13.41)	62 (17.77)	110 (30.81)	<0.001
Ventilator	980 (69.50)	217 (62.72)	235 (65.64)	246 (70.49)	282 (78.99)	<0.001
CRRT	46 (3.26)	2 (0.58)	4 (1.12)	7 (2.01)	33 (9.24)	<0.001
Cerebral Surgery	158 (11.21)	51 (14.74)	33 (9.22)	42 (12.03)	32 (8.96)	0.050
Outcomes						
Hospital stay (day)	8.57 (4.18, 16.13)	8.30 (3.94, 16.10)	7.64 (4.44, 15.05)	8.75 (3.91, 14.86)	10.11 (4.77, 19.37)	0.011
ICU stay (day)	3.92 (1.88, 8.62)	3.88 (1.77, 9.58)	3.83 (1.90, 7.43)	3.65 (1.87, 8.28)	4.46 (1.97, 9.61)	0.233
90-day mortality, *n* (%)	501 (35.53)	76 (21.97)	93 (25.98)	138 (39.54)	194 (54.34)	<0.001
365-day mortality, *n* (%)	601 (42.62)	103 (29.77)	109 (30.45)	167 (47.85)	222 (62.18)	<0.001

RAR, red blood cell distribution width to albumin ratio; SBP, Systolic Blood Pressure; DBP, Diastolic Blood Pressure; SpO2, oxygen saturation; SOFA, sequential organ failure assessment; GCS, Glasgow coma scale; LODS, Logistic Organ Dysfunction System; AKI, acute kidney injury; WBC, white blood cell; BUN, blood urea nitrogen; INR, international normalized ratio; PT, prothrombin time; CRRT, continuous renal replacement therapy; ICU, intensive care unit.

Participants were grouped based on RAR quartiles as follows: Quartile (Q) 1:<3.22%/(g/dL); Q2: 3.22–3.66%/(g/dL); Q3: 3.66–4.39%/(g/dL); Q4: ≥4.39%/(g/dL). Table 2 displays the baseline characteristics and clinical outcomes across these quartiles. Individuals in the highest RAR quartile (Q4) exhibited significantly higher heart rate, respiratory rate, BUN, creatinine, potassium, international normalized ratio (INR), and prothrombin time, while having significantly lower blood pressure, platelet count, and sodium levels. Additionally, Q4 patients had higher SOFA and LODS scores, indicating greater disease severity. In terms of comorbidities, patients in Q4 showed a greater prevalence of MI, Congestive heart failure, AKI and sepsis, but a lower prevalence of hypertension. They were also more likely to receive diuretic, vasoactive drug, ventilator, and CRRT. Higher RAR levels were associated with longer hospital stays (*p* < 0.05). Mortality rates were significantly elevated among individuals in the highest RAR quartile: 90-day (54.34% vs. 39.54, 25.98, and 21.97%; *p* < 0.001) and 365-day mortality (62.18% vs. 47.85, 30.45, and 29.77%; *p* < 0.001).

Supplementary Table S2 outlines the baseline differences between survivors and non-survivors at 365 days. Notably, non-survivors exhibited a higher RAR compared to survivors (4%/(g/dL) vs. 3.47%/(g/dL), *p* < 0.001). Additionally, non-survivors were likely to be older and exhibited higher diastolic blood pressure, respiratory rate, and oxygen saturation, SOFA and LODS scores. They also showed a greater prevalence of congestive heart failure, AKI, and sepsis. Laboratory findings revealed that non-survivors had higher WBC, platelets, BUN, creatinine, potassium, glucose, INR, anion gap and prothrombin time. In terms of treatment, non-survivors received more diuretics, mannitol, vasoactive drugs, ventilator support, CRRT, and cerebral surgery, but were less likely to receive statins.

### Primary outcomes

**Figure 2:**
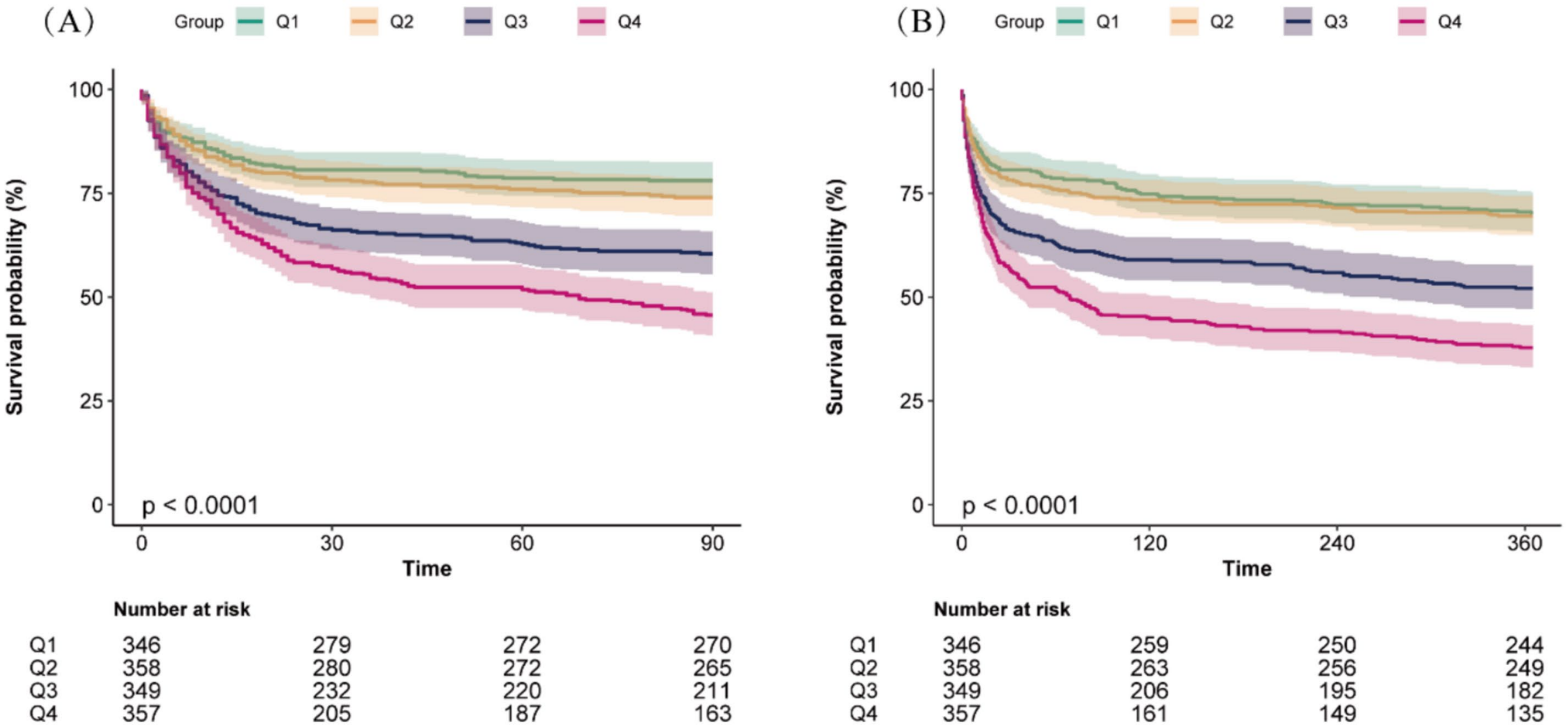
Kaplan–Meier survival analysis curves for all-cause mortality at 90-day **(A)** and 360-day **(B)** across different red blood cell distribution width to albumin ratio (RAR) quartiles.

Kaplan–Meier survival analysis curves were employed to analyze incidence of 90-day and 360-day mortality across RAR quartiles, as shown in Figure 2. Individuals in the highest RAR quartile had a lower survival rate at both the 90-day and 360-day time points (log-rank *p* < 0.001).

**Figure 3:**
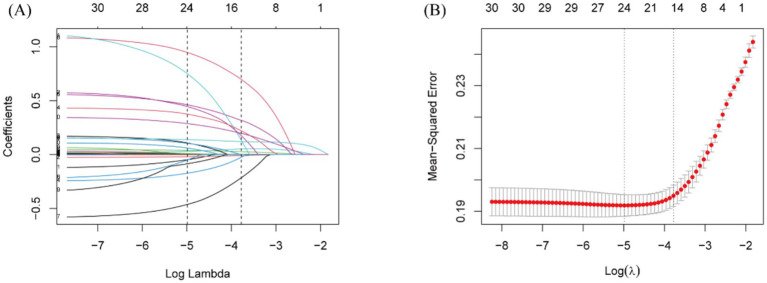
Lasso regression-based variable screening. **(A)** Variation characteristics of variable coefficients. **(B)** The process of selecting the optimal value of the parameter *λ* in the lasso regression model is carried out by the cross-validation method.

To identify risk factors for 365-day all-cause mortality, LASSO regression was conducted, with tenfold cross-validation used for iterative analysis (Figure 3). A total of 15 variables were identified as closely associated with 365-day mortality, including RAR, age, race, SOFA score, LODS score, sepsis, BUN, potassium, glucose, vasoactive drug use, statin use, mannitol use, CRRT, cerebral surgery, and length of hospital stay. These variables were considered potential confounders and were incorporated into multivariable models predicting mortality.

Time-varying Cox regression analysis (Table 3) demonstrated a significant link between RAR and the risk of death at both 90 and 365 days. When RAR was analyzed as a numeric predictor, the hazard ratios (HRs) regarding 365-day mortality were as follows: model 1: HR: 1.14 (95%CI: 1.10 ~ 1.17, P<0.001), model 2: HR, 1.11 (95%CI: 1.08 ~ 1.15, *p* < 0.001), and model 3: HR:1.07 (95%CI: 1.02 ~ 1.13, *p* = 0.005). In the case of RAR as a nominal variable, individuals in Q4 exhibited a markedly elevated 365-day mortality risk relative to those in Q1: model 1: HR 2.62 (95% CI: 1.99 ~ 3.46, *p* < 0.001), model 2: HR 2.46 (95% CI: 1.87 ~ 3.24, *p* < 0.001), and model 3: HR 1.67 (95% CI 1.24 ~ 2.25, *p* = 0.001). A similar trend was identified through the time-varying Cox regression analysis of RAR and 90-day mortality.

**Table 3:** Time-varying Cox regression model for all-cause mortality.

Variable	Model 1		Model 2		Model 3	
	HR (95%CI)	*p*-value	HR (95%CI)	*p*-value	HR (95%CI)	*p*-value
90-day mortality						
RAR (per unit)	1.11 (1.04 ~ 1.19)	0.001	1.10 (1.03 ~ 1.17)	0.003	1.14 (1.05 ~ 1.22)	0.001
RAR (quartiles)						
Q1 (RAR < 3.22)	1 (ref)		1 (ref)		1 (ref)	
Q2 (3.22 ≤ RAR < 3.66)	1.61 (0.77 ~ 3.38)	0.206	1.63 (0.78 ~ 3.41)	0.195	1.77 (0.84 ~ 3.75)	0.132
Q3 (3.66 ≤ RAR < 4.39)	1.74 (0.87 ~ 3.48)	0.115	1.75 (0.88 ~ 3.50)	0.113	1.79 (0.89 ~ 3.60)	0.104
Q4 (4.39 ≤ RAR)	3.18 (1.66 ~ 6.09)	<0.001	3.17 (1.65 ~ 6.06)	<0.001	3.65 (1.89 ~ 7.06)	<0.001
*P* for trend		<0.001		<0.001		<0.001
365-day mortality						
RAR (per unit)	1.14 (1.10 ~ 1.17)	<0.001	1.11 (1.08 ~ 1.15)	<0.001	1.07 (1.02 ~ 1.13)	0.005
RAR (quartiles)						
Q1 (RAR < 3.22)	1 (ref)		1 (ref)		1 (ref)	
Q2 (3.22 ≤ RAR < 3.66)	1.13 (0.82 ~ 1.55)	0.459	1.05 (0.76 ~ 1.44)	0.764	1.02 (0.74 ~ 1.40)	0.911
Q3 (3.66 ≤ RAR < 4.39)	1.85 (1.38 ~ 2.47)	<0.001	1.66 (1.24 ~ 2.23)	0.001	1.35 (1.00 ~ 1.82)	0.05
Q4 (4.39 ≤ RAR)	2.62 (1.99 ~ 3.46)	<0.001	2.46 (1.87 ~ 3.24)	<0.001	1.67 (1.24 ~ 2.25)	0.001
*P* for trend		<0.001		<0.001		<0.001

Model 1: unadjusted.

Model 2: adjusted for age, gender, race.

Model 3: adjusted for Model 2 plus SOFA, LODS, sepsis, BUN, Potassium, Glucose, Vasoactive drug, Statin, Mannitol, CRRT, Cerebral surgery, Length of stay in hospital.

RAR, red blood cell distribution width to albumin ratio; SOFA, sequential organ failure assessment; LODS, Logistic Organ Dysfunction System; BUN, blood urea nitrogen; CRRT, continuous renal replacement therapy.

**Figure 4:**
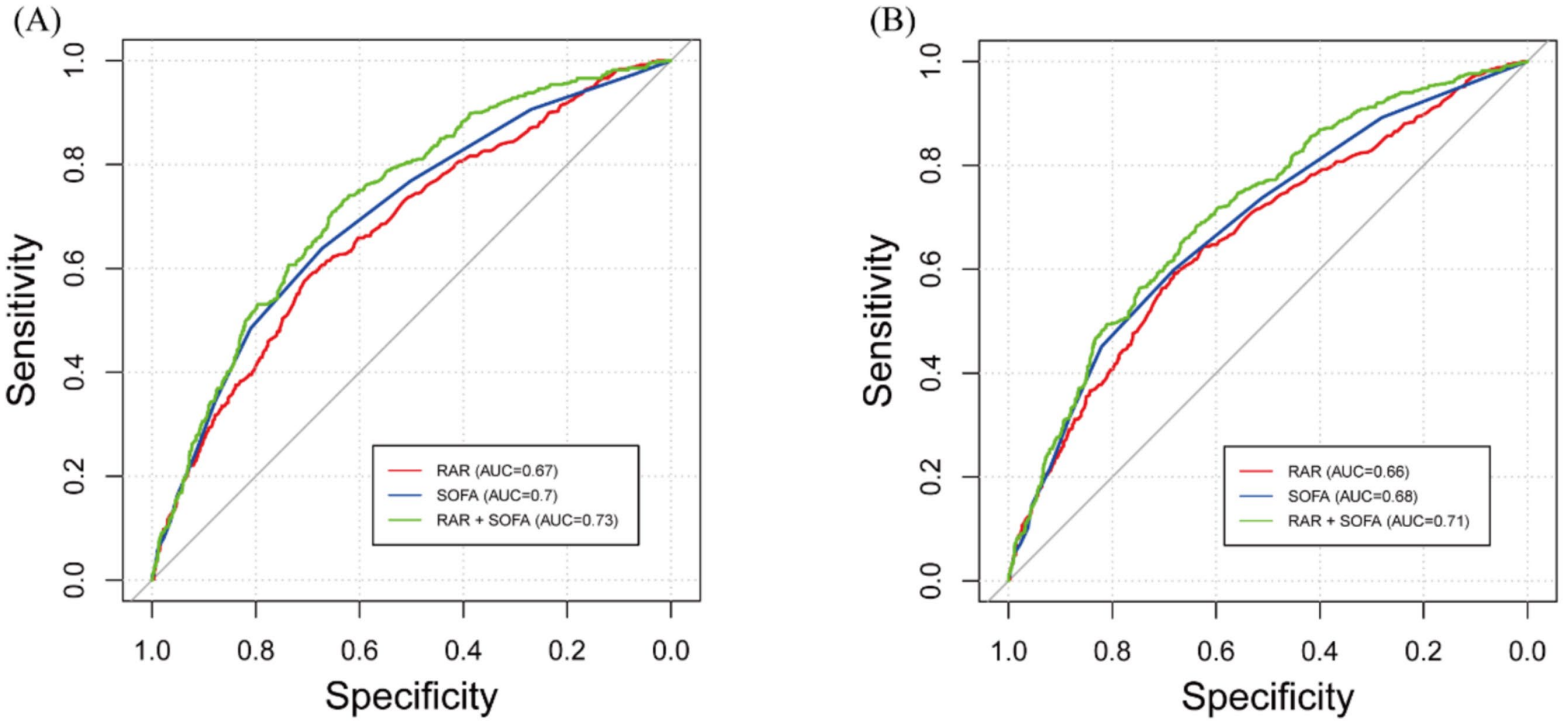
Receiver operating characteristic (ROC) curves for predicting all-cause mortality. **(A)** 90-day all-cause mortality. **(B)** 365-day mortality. RAR, red blood cell distribution width to albumin ratio; SOFA, and their combination are shown.

To evaluate the prognostic predictive ability of RDW, albumin, and the composite RAR, we performed ROC curve analysis for both 90-day and 365-day mortality (Table 4; Supplementary Figure S2). In addition, we evaluated the predictive performance of the SOFA score and the combination of RAR and SOFA score (Figure 4; Table 4).

**Table 4:** The predictive performance of RAR on all-cause mortality.

Variables	Cut-off	Sensitivity	Specificity	PPV	NPV	AUC (95%CI)
90-day mortality						
RDW	13.8	0.655	0.546	0.443	0.741	0.627 (0.597, 0.695)
Albumin	3.75	0.641	0.629	0.500	0.645	0.666 (0.636, 0.703)
RAR	3.9	0.585	0.697	0.516	0.753	0.674 (0.645, 0.703)
SOFA	3.5	0.639	0.672	0.518	0.771	0.699 (0.671, 0.728)
RAR + SOFA		0.758	0.603	0.513	0.819	0.730 (0.703, 0.757)
365-day mortality						
RDW	13.6	0.704	0.492	0.507	0.691	0.626 (0.596, 0.655)
Albumin	5.35	0.002	0.999	0.500	0.574	0.652 (0.624, 0.681)
RAR	3.8	0.592	0.680	0.579	0.692	0.663 (0.634, 0.692)
SOFA	3.5	0.599	0.681	0.583	0.696	0.683 (0.656, 0.711)
RAR + SOFA		0.717	0.606	0.575	0742	0.714 (0.687, 0.740)

RAR, red blood cell distribution width to albumin ratio; RDW, red blood cell distribution width; SOFA, sequential organ failure assessment; PPV, positive predictive value; NPV, negative predictive value; AUC, area under the curve.

For 90-day mortality, the optimal cutoff for RDW was 13.8, with an AUC of 0.627 (95% CI: 0.597–0.695), for albumin it was 3.75 with an AUC of 0.666 (95% CI: 0.636–0.703), and for RAR it was 3.9 with an AUC of 0.674 (95% CI: 0.645–0.703). The optimal cutoff for SOFA was 3.5, with an AUC of 0.699 (95% CI: 0.671–0.728), and the combination of RAR and SOFA improved the AUC to 0.730 (95% CI: 0.703–0.757). DeLong’s test showed that RAR had a significantly higher AUC than RDW (*p* < 0.001), and the combination of RAR and SOFA had a significantly higher AUC than either RAR or SOFA alone (*p* < 0.001 for both comparisons). However, the AUC for RAR was not significantly different from albumin (*p* = 0.6947).

For 365-day mortality, the optimal cutoff for RDW was 13.6 with an AUC of 0.626 (95% CI: 0.596–0.655), for albumin it was 5.35 with an AUC of 0.652 (95% CI: 0.624–0.681), and for RAR it was 3.8 with an AUC of 0.663 (95% CI: 0.634–0.692). The optimal cutoff for SOFA was 3.5, with an AUC of 0.683 (95% CI: 0.656–0.711), and the combination of RAR and SOFA resulted in an improved AUC of 0.714 (95% CI: 0.687–0.740). DeLong’s test revealed that RAR had a significantly higher AUC than RDW (*p* = 0.001), and the combination of RAR and SOFA significantly improved predictive performance compared to RAR or SOFA alone (*p* < 0.001 for both comparisons). However, the AUC for RAR was not significantly different from albumin (*p* = 0.608). To further assess the clinical value of RAR, we conducted DCA, which showed that the SOFA and RAR model provided greater net benefit than the SOFA-alone model across a wide range of thresholds for both 90-day and 365-day mortality (Supplementary Figure S3).

In the fully adjusted models, RAR demonstrated superior predictive performance over RDW for both 90-day and 365-day mortality. The AUC for 90-day mortality increased from 0.822 with RDW to 0.831 with RAR (ΔAUC = 0.009, *p = 0.004*), with corresponding NRI and IDI of 3.55% (*p* = 0.018) and 1.17% (*p* < 0.001), respectively. Similar improvements were observed for 365-day mortality (ΔAUC = 0.008, *p* = 0.017; NRI = 3.67%, *p* = 0.022; IDI = 1.16%, *p* < 0.001). However, no statistically significant difference was found between RAR and albumin in AUC, NRI, or IDI, indicating comparable overall discriminative ability (Supplementary Table S5).

**Figure 5:**
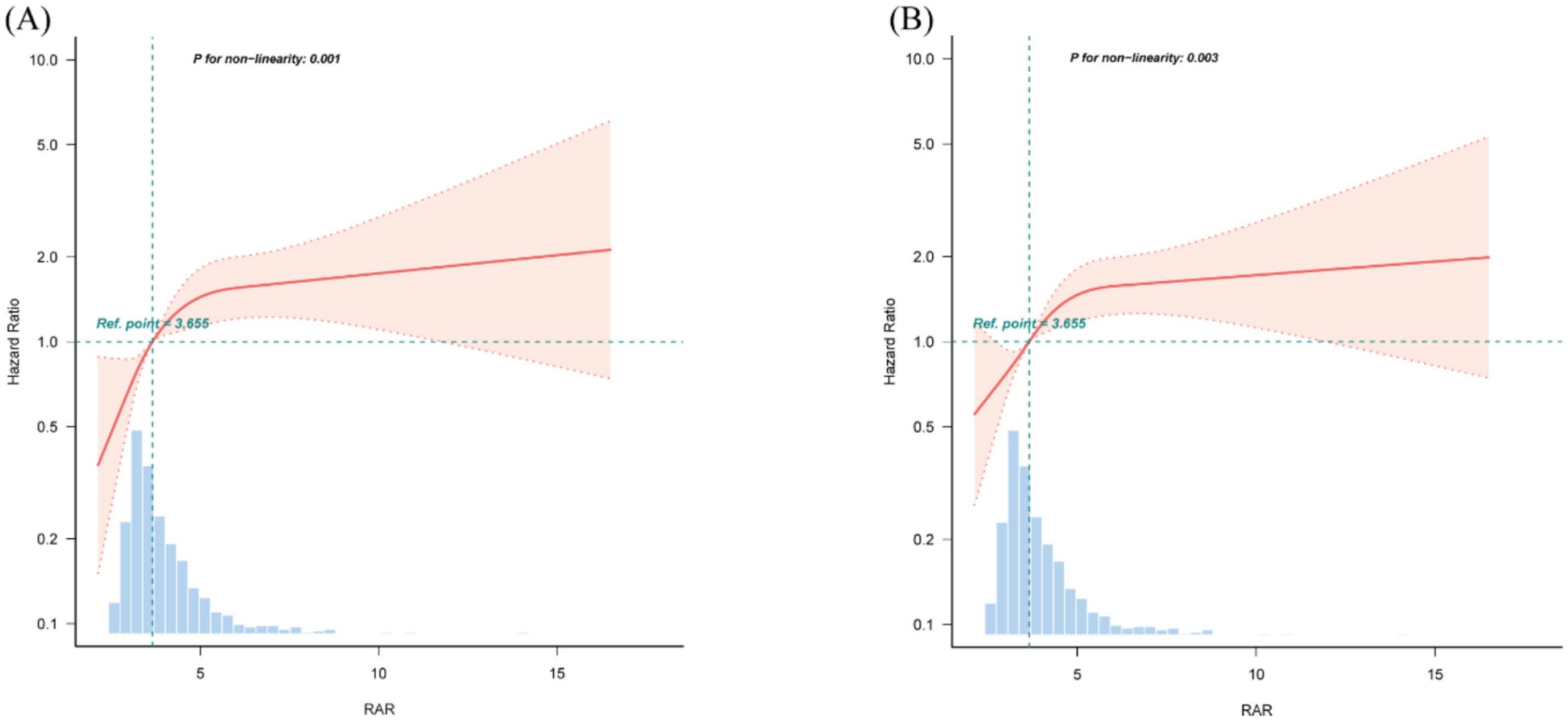
Restricted cubic spline (RCS) curves for RAR and hazard ratios. **(A)** 90-day mortality. **(B)** 365-day mortality. The median value of RAR serves as the reference. Models are adjusted for all covariates in Model 3 (Table 3). RAR, red blood cell distribution width to albumin ratio.

Furthermore, RCS analyses presented in Figure 5 demonstrated a non-linear correlation between higher RAR and mortality at both 90 and 365 days, which persisted after adjustment for relevant covariates (P for non-linearity = 0.001 and 0.003, respectively). To further explore this non-linear relationship, we performed a two-piecewise linear regression analysis. This revealed significant infection points for RAR, which were found to be 4.82 for 90-day mortality and 4.73 for 365-day mortality (Supplementary Table S6). Below these thresholds, RAR was significantly associated with increased mortality risk, with HR of 1.41 (95% CI: 1.14–1.73) for 90-day mortality and 1.37 (95% CI: 1.13–1.66) for 365-day mortality. However, when RAR values exceeded these thresholds, the mortality risk remained relatively stable, with HRs close to 1.00.

### Subgroup analyses

**Figure 6:**
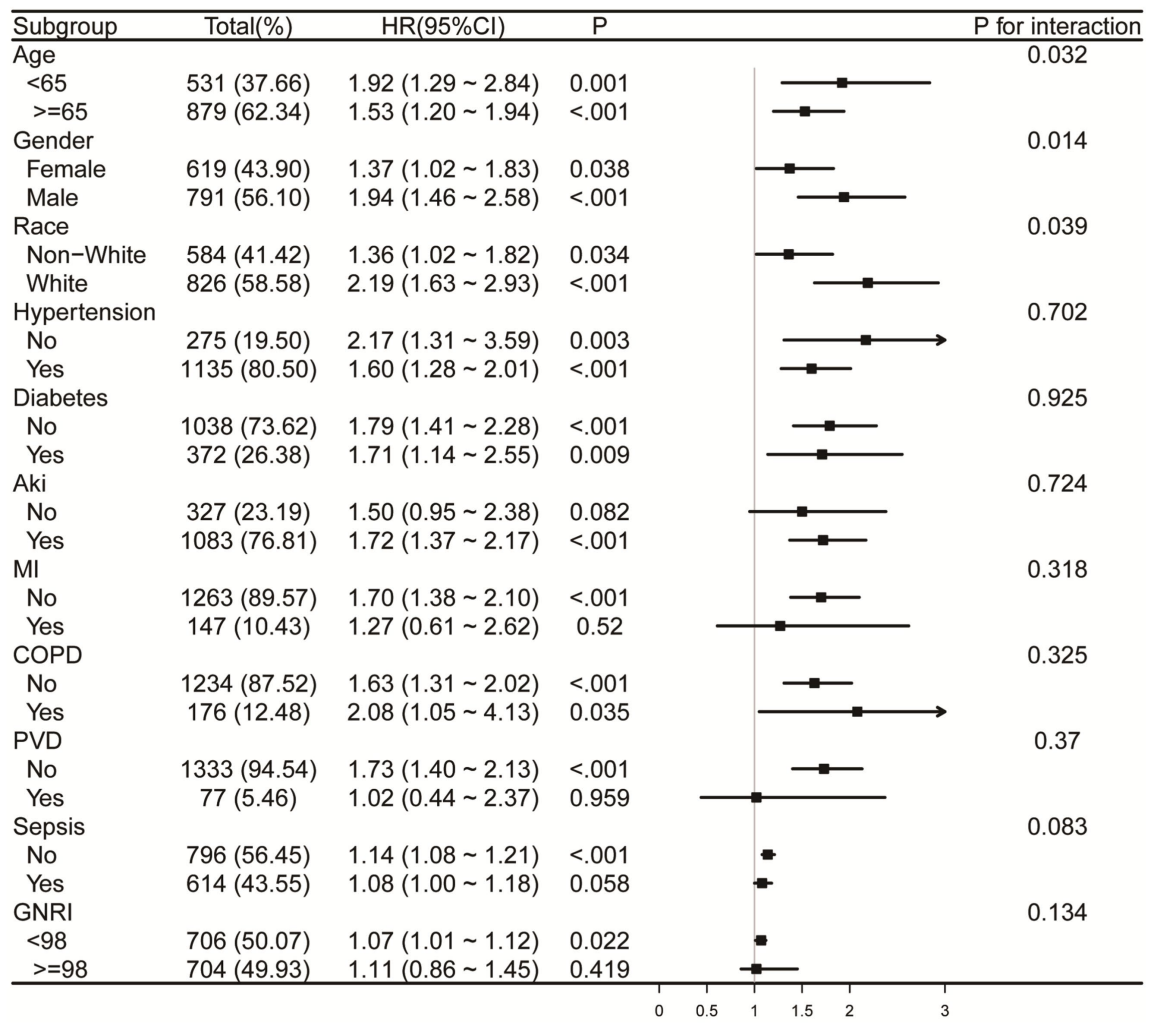
Forest plots for subgroup analysis of RAR and 90-day all-cause mortality, adjusted for covariates in Model 3 (Table 3), except the stratification variable. RAR, red blood cell distribution width to albumin ratio; AKI, acute kidney injury; MI, myocardial infarction. COPD, chronic pulmonary disease; PVD, peripheral vascular disease; GNRI, geriatric nutrition risk index.

**Figure 7:**
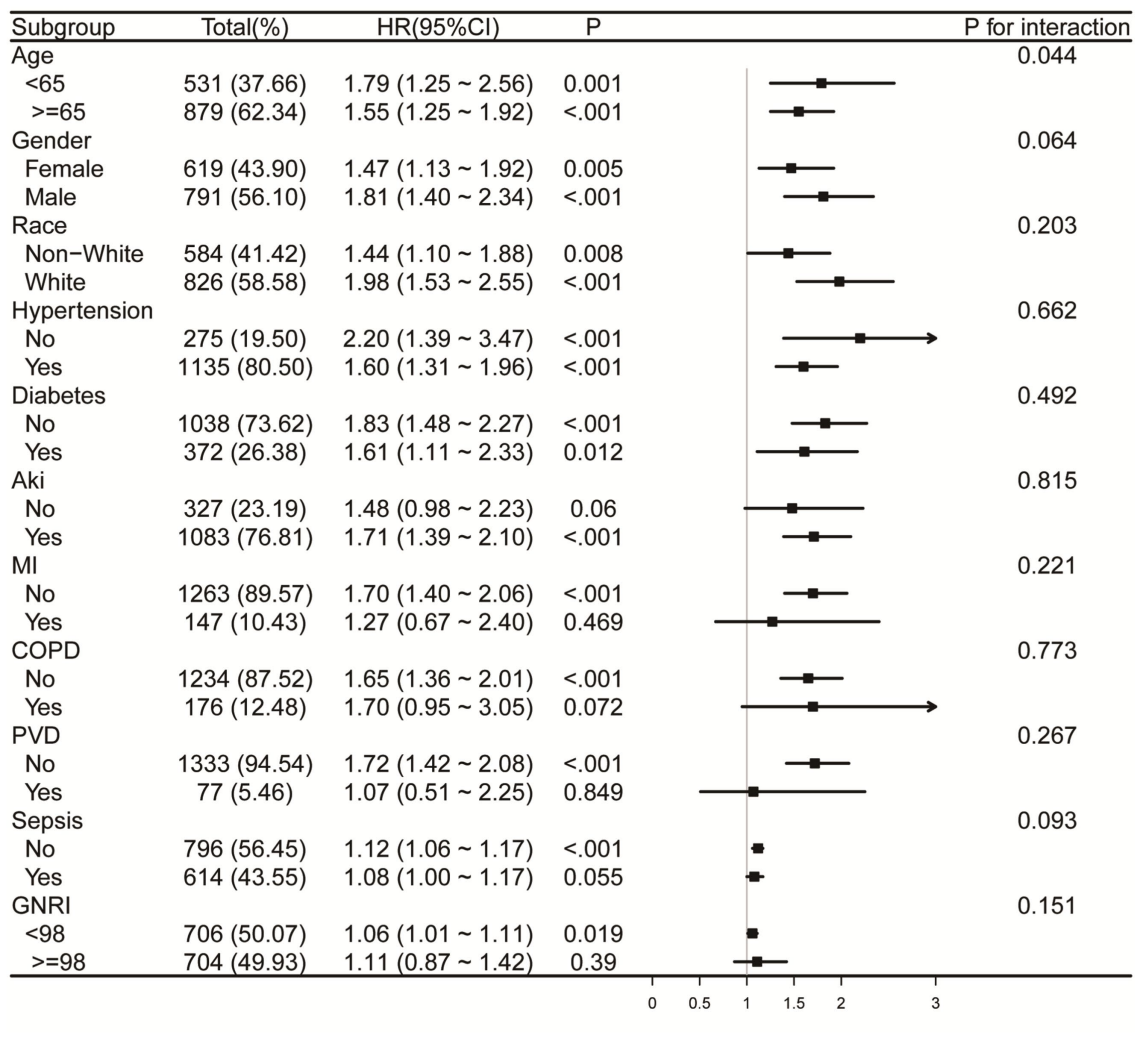
Forest plots for subgroup analysis of RAR and 365-day all-cause mortality, adjusted for covariates in Model 3 (Table 3), except the stratification variable. RAR, red blood cell distribution width to albumin ratio; AKI, acute kidney injury; MI, myocardial infarction. COPD, chronic pulmonary disease; PVD, peripheral vascular disease; GNRI, geriatric nutrition risk index.

Forest plots (Figures 6, 7) illustrate the relationship between RAR and the risk of death in various subgroups of ICH patients. Stratification by age (<65 or ≥65 years), gender, race, hypertension, diabetes, AKI, MI, COPD, PVD, sepsis, and GNRI (<98 or ≥98) revealed a largely uniform effect of RAR across different subpopulations (P for interaction > 0.05), with the exception of age (P for interaction = 0.032), gender (P for interaction = 0.014), and race (P for interaction = 0.039). Patients aged <65 years with elevated RAR exhibited an increased mortality risk at 90 and 365 days compared to their counterparts. Similarly, male patients demonstrated significantly greater 90-day mortality than their female counterparts. Regarding racial differences, White patients appeared to have a higher all-cause mortality risk at 90 days compared to individuals from other racial groups. Additionally, we found that in both the sepsis group and the GNRI ≥98 group, there was no significant relationship between RAR and mortality (*p* > 0.05).

### Sensitivity analysis

To validate the imputation procedure, we compared the distributions of key variables before and after imputation. Supplementary Figure S1 compares the original and imputed datasets, with non-missing values shown in blue and imputed values in red. The substantial overlap between the two indicates high-quality imputation.

In addition, we performed sensitivity analyses using a complete-case dataset, excluding any individuals with missing data. Time-varying Cox regression analysis were repeated in this subset, and the results were consistent with those obtained from the imputed dataset in terms of direction and statistical significance (Supplementary Table S7). These findings indicate that our main conclusions are robust to the handling of missing data.

## Discussion

This research represents the first comprehensively assessment of RAR’s prognostic relevance in ICH patients using a large-scale cohort and robust statistical methodologies. The results of this study show that higher RAR levels are significantly linked to increased overall mortality at both 90 and 365 days, independent of established risk factors.

RDW serves as a hematological indicator that captures differences in erythrocyte size, commonly referred to as anisocytosis. Elevated RDW levels indicate increased erythrocyte heterogeneity, which may result from systemic inflammation, oxidative stress, nutritional deficiencies, and impaired erythropoiesis (5). Recent investigations have highlighted the predictive value associated with RDW among ICH individuals. Xu et al. reported that higher RDW at admission were significantly linked to a greater all-cause mortality risk at 3 months (odds ratio [OR]: 2.88, 95%CI: 0.96–8.64) and at 1 year (OR: 3.16, 95% CI: 1.08–9.21) (12). Comparable results were found in the researches conducted by He et al. and Pinho et al. (13, 14). Cui et al. proposed a nomogram integrating RDW to predict the likelihood of adverse 30-day outcomes in individuals with ICH. RDW was identified as a key laboratory indicator influencing prognosis. Calibration plots demonstrated strong consistency between predicted and actual outcomes, supporting the model’s robustness, whereas decision curve analysis highlighted greater net gain in clinical decision-making, reinforcing its usefulness in practice (15). Albumin, synthesized by the liver, is the most abundant protein in human plasma and plays a crucial role in maintaining colloidal osmotic pressure, antioxidant activity, and anti-inflammatory responses. Serum albumin levels are widely recognized as an indicator of nutritional status, hepatic function, and systemic inflammation. Decreased plasma albumin levels have been recognized as a distinct risk indicator for multiple critical illnesses and are linked to adverse health outcomes (16–18). Shang et al. carried out a retrospective investigation involving 229 cases of subarachnoid hemorrhage and found that, compared to the high-albumin group, patients with low serum albumin levels had an 18.51 times higher risk of adverse outcomes (OR: 18.51, 95% CI: 3.41–349.03). Additionally, hospital length of stay and ICU admission duration were significantly prolonged in the low-albumin group (18). In light of these results, we propose that albumin may also function as an important prognostic biomarker for evaluating illness intensity and forecasting patient prognosis in ICH.

Due to the complexity of ICH and its clinical progression, which is influenced by multiple factors, relying on a single predictive marker is often inadequate for accurately evaluating patient outcomes. The RAR, which integrates RDW and serum albumin, addresses this limitation by providing a more comprehensive evaluation, thereby enhancing prognostic accuracy in ICH patients. Recent researches have demonstrated the prognostic value of RAR across various clinical conditions beyond ICH. Hao et al., in a prospective study, demonstrated that elevated RAR was linked to greater risk of death in the general population and in various diseases, such as malignancies and cardiovascular conditions (19). Similarly, a retrospective analysis by Cai et al. of 612 post-cardiac arrest patients revealed that higher RAR levels were linked to evaluated 30-day and 180-day mortality risks, with RAR demonstrating superior predictive value compared to RDW or albumin alone (20). Among severely ill patients suffering from coronary heart disease and diabetes, increased RAR was correlated with increased risk of mortality in both early and long-term mortality, exhibiting a linear relationship with one-year mortality (21). Additionally, Zhou et al. analyzed 2,077 individuals with non-ischemic heart failure and found that each log2 increase in RAR was significantly linked to a 2.329-fold increased risk of mortality or heart transplantation (HR: 2.329, 95% CI: 1.677–3.237, *p* < 0.001). Incorporating RAR into traditional prognostic models significantly improved model discrimination, calibration, and reclassification capacity (*p* < 0.001 for all) (22). Furthermore, elevated RAR levels have been linked to in-hospital and one-year mortality in acute severe pulmonary embolism (*p* < 0.05) have also demonstrated prognostic value in individuals affected by COVID-19 and various other conditions (23–25). In our retrospective study of 3,148 ICH patients, we found that an increase in RAR corresponded with increased 90-day and 365-day all-cause mortality risks. RCS analysis also demonstrated a non-linear trend in the relationship between RAR and death risk. To further explore this relationship, we performed a two-piecewise linear regression analysis, which identified the infection points of 4.82 for 90-day mortality and 4.73 for 365-day mortality. This suggests that RAR may serve as an effective prognostic biomarker in patients with values below these thresholds. Specifically, lower RAR levels appear to be strongly associated with an increased risk of death, which may allow clinicians to identify high-risk patients who could benefit from more intensive monitoring or early interventions. However, above these threshold values, the predictive value of RAR diminishes. This suggests that RAR may not be as useful for predicting mortality in patients with higher RAR values. In such cases, other prognostic biomarkers or clinical factors may be needed to further refine risk stratification and guide treatment decisions.

The mechanisms linking increased RAR with poor prognosis in individuals with ICH remain insufficiently understood and may be attributed to several potential pathophysiological mechanisms. First, elevated RDW reflects increased erythrocyte heterogeneity, often driven by systemic inflammation. Proinflammatory cytokines, particularly tumor necrosis factor-alpha (TNF-*α*), impair erythropoiesis and reduce erythrocyte survival, thereby contributing to anisocytosis (26). Several studies have indicated a close correlation between RDW and circulating TNF-α levels among critically ill patients (27). As an early-stage cytokine, elevated TNF-α levels may contribute to blood–brain barrier disruption, cerebral edema, and neuronal apoptosis, which are hallmark features of secondary brain injury and have been associated with increased mortality in ICH patients (28). Additionally, systemic inflammation and metabolic stress are increasingly recognized contributors to poor outcomes across acute vascular diseases (29, 30), further supporting the pathological significance of inflammation-related markers such as RDW. Second, Rodríguez-Carrio et al. showed that increased RDW is associated with depletion of endothelial progenitor cells and elevated vascular inflammatory mediators, indicating a mechanistic link between RDW and endothelial dysfunction, a central contributor to cerebral microvascular injury in ICH (31). Third, elevated RDW has also been associated with heightened oxidative stress, which plays a key role in aggravating brain injury and has been linked to worse outcomes and higher mortality in ICH patients (32, 33). Furthermore, increased RDW is related to impaired erythrocyte deformability and enhanced platelet–endothelial interactions, both of which may exacerbate secondary bleeding and vascular injury in the acute phase of ICH (34, 35). In parallel, hypoalbuminemia reflects a catabolic, pro-inflammatory state and diminished antioxidant defense (36). Zhou et al. highlighted that inflammation and oxidative stress are major contributors to secondary brain injury in ICH, leading to blood–brain barrier disruption, cerebral edema, and neuronal apoptosis (11). Albumin plays a protective role by modulating oncotic pressure, neutralizing free radicals, and stabilizing endothelium. Therefore, low albumin levels may further increase vascular permeability and promote cerebral edema. In addition, Shang et al. reported that lower albumin levels significantly predicted adverse outcomes in patients with subarachnoid hemorrhage, a condition with overlapping pathophysiology with ICH (18). This highlights the prognostic value of albumin in hemorrhagic cerebrovascular disorders. Taken together, an elevated RAR may serve as a composite surrogate marker of systemic inflammation, oxidative imbalance, and poor vascular integrity—all of which contribute to secondary injury and increased mortality in ICH. This mechanistic insight reinforces the clinical relevance of RAR as a prognostic biomarker.

Furthermore, this study evaluates the predictive capability of RAR for all-cause mortality in ICH patients. Our findings indicate that RAR’s predictive ability for all-cause mortality is comparable to albumin but outperforms RDW alone. Although RAR and albumin demonstrated comparable AUCs for mortality prediction, combining RDW and albumin into a single ratio offers both physiological and practical advantages. Serum albumin, although widely used, is susceptible to fluctuations caused by fluid resuscitation, hepatic dysfunction, and acute-phase responses, which may limit its reliability as an independent prognostic marker. In contrast, RDW reflects systemic inflammation, oxidative stress, and bone marrow dysfunction, which are important prognostic factors not captured by albumin. By integrating these two biologically distinct parameters, RAR provides a more comprehensive assessment of the patient’s underlying pathophysiological condition. This broader coverage of disease mechanisms may enhance its utility as a prognostic indicator beyond the capacity of either RDW or albumin alone. The integration of RAR with SOFA improved the predictive performance at both time points, suggesting that combining biomarkers with clinical scores can provide a more robust model for mortality prediction in ICH. The DCA results further showed that the SOFA + RAR model offered greater net clinical benefit than SOFA alone across a broad range of threshold probabilities, highlighting RAR’s potential as an accessible biomarker for early risk stratification in clinical settings.

Moreover, our subgroup analyses reveal novel insights into the differential impact of RAR on mortality across age, sex, and racial groups, underscoring the importance of individualized prognostic assessments. Regarding gender differences, male patients tend to have a higher baseline level of systemic inflammation and may experience different hormonal regulation, which could contribute to the stronger association between RAR and mortality in this group. For example, estrogen has anti-inflammatory effects, potentially explaining why women may exhibit a different inflammatory profile compared to men (37, 38). Additionally, racial differences in mortality risk may reflect a combination of genetic factors, socio-economic factors, and access to healthcare, which may explain why White patients showed a higher mortality risk associated with elevated RAR (39). The stronger association between RAR and mortality in patients aged <65 years may be explained by several factors. Younger patients tend to have a more robust immune response, which may lead to more rapid clinical deterioration when faced with high levels of RAR, potentially due to increased inflammation, coagulation dysfunction, and vascular permeability. In contrast, older patients (≥65 years) might have developed compensatory mechanisms to cope with chronic inflammation, oxidative stress, and other age-related conditions, making them less susceptible to the detrimental effects of elevated RAR. Additionally, we found that in both the sepsis group and the GNRI ≥98 group, there was no significant relationship between RAR and mortality. This lack of significant association may be explained by the relatively stable immune and nutritional statuses of individuals in these groups. In the sepsis group, despite the presence of infection, it is possible that the immune response or therapeutic interventions in these patients attenuated the inflammatory response, thereby reducing the variability in RAR levels and diminishing the association with mortality. Similarly, individuals in the GNRI ≥98 group, who generally exhibit better nutritional status, show smaller variations in RAR, which likely accounts for the lack of significant correlation with mortality outcomes.

Despite these strengths, our investigation is subject to several constraints. First, given its retrospective and observational nature, causal relationships cannot be definitively established. Additionally, the exclusion of certain confounders due to missing data may introduce potential limitations despite multivariable adjustments, as this could affect both the accuracy and generalizability of our results. Second, while our findings were derived from a large, well-characterized cohort, the use of a single-center database may introduce potential selection bias, which limits the generalizability of the results. External validation in prospective multicenter studies is required to establish the broader applicability of the RAR in prognostic assessment for patients with ICH. Third, the lack of detailed information on the dynamic changes in RAR over time limits our ability to assess its temporal trajectory in relation to disease progression. Future research should explore the mechanistic pathways linking RAR to ICH outcomes and evaluate its clinical utility in guiding therapeutic strategies. Lastly, although we propose potential mechanisms linking the RAR with inflammation and malnutrition, our study does not include direct measurements of cytokine levels or nutritional assessments, which limits the validation of these mechanisms. Future research should include cytokine levels and detailed nutritional assessments to further validate these hypotheses and gain a more comprehensive understanding of the relationship between RAR and ICH outcomes.

## Conclusion

In conclusion, our study shows that an elevated RAR is significantly associated with increased mortality in ICH patients. Combined with the SOFA score, RAR enhances prognostic accuracy, helping identify high-risk patients for early intervention. The non-linear relationship between RAR and mortality allows for personalized risk stratification, supporting RAR as a valuable and cost-effective biomarker for improving risk assessment and clinical decision-making in ICH.

## Data Availability

Publicly available datasets were analyzed in this study. This data can be found at: https://mimic.mit.edu/ Medical Information Mart for Intensive Care (MIMIC)-IV Certification ID: 64822128.
