# Supplementary material for: Association between red blood cell distribution width-to-albumin ratio and all-cause mortality in intracerebral hemorrhage
Source: Front Nutr. 2025 Jun 4;12:1599104. doi: 10.3389/fnut.2025.1599104 (PMC12175621; doi:10.3389/fnut.2025.1599104)
Supplement: Supplementary file 1 [file Table_1.docx]

Table S1 missing number of variables.

| **Variables** | **Miss.freq** | **Miss.percentage** |
| --- | --- | --- |
| Age | 0 | 0 |
| Gender | 0 | 0 |
| Race | 0 | 0 |
| Heart rate | 0 | 0 |
| SBP | 0 | 0 |
| DBP | 0 | 0 |
| Temperature | 6 | 0.0043 |
| Respiratory rate | 2 | 0.0014 |
| SpO2 | 1 | 0.0007 |
| SOFA | 0 | 0 |
| GCS | 0 | 0 |
| LODS | 0 | 0 |
| Congestive heart failure | 0 | 0 |
| Hypertension | 0 | 0 |
| Diabetes | 0 | 0 |
| AKI | 0 | 0 |
| Chronic pulmonary diseas | 0 | 0 |
| Peripheral vascular disease | 0 | 0 |
| Myocardial infarction | 0 | 0 |
| Sepsis | 0 | 0 |
| WBC | 0 | 0 |
| neutrophils | 917 | 0.65 |
| lymphocytes | 915 | 0.65 |
| Platelet | 2 | 0.0014 |
| total bilirubin | 392 | 0.278 |
| CRP | 1244 | 0.882 |
| TG | 629 | 0.446 |
| LDL | 1386 | 0.982 |
| HDL | 700 | 0.496 |
| cholesterol | 696 | 0.493 |
| HbA1c | 672 | 0.476 |
| BUN | 4 | 0.0028 |
| Creatinine | 5 | 0.0035 |
| Potassium | 5 | 0.0035 |
| Sodium | 5 | 0.0035 |
| Glucose | 6 | 0.0043 |
| INR | 94 | 0.0667 |
| Anion gap | 6 | 0.0043 |
| PT | 93 | 0.0660 |
| Beta blockers | 0 | 0 |
| Diuretic | 0 | 0 |
| Mannitol | 0 | 0 |
| Statin | 0 | 0 |
| Vasoactive drug | 0 | 0 |
| Ventilator | 0 | 0 |
| CRRT | 0 | 0 |
| Cerebral Surgery | 0 | 0 |

Abbreviations: SBP, Systolic Blood Pressure; DBP, Diastolic Blood Pressure; SpO2, oxygen saturation;SOFA, sequential organ failure assessment; GCS, Glasgow coma scale; LODS, Logistic Organ Dysfunction System; AKI, acute kidney injury; WBC, white blood cell; CRP, C-reactive protein; TG, triglycerides; LDL: Low-Density Lipoprotein; HDL: High-Density Lipoprotein; HbA1c: Hemoglobin A1c; BUN, blood urea nitrogen; INR, international normalized ratio; PT, prothrombin time; CRRT, continuous renal replacement therapy.

TableS2 Baseline characteristics between 365-day survival and 365-day mortality group

| **Variables** | **Total**  **(n = 1410)** | **365-day survival**  **(n = 809)** | **365-day mortality**  **(n = 601)** | **P-value** |
| --- | --- | --- | --- | --- |
| RAR[%/(g/dL)] | 3.66 (3.22, 4.39) | 3.47 (3.14, 4.03) | 4.00 (3.42, 4.82) | <0.001 |
| Age (year) | 69.00 (58.00, 80.00) | 67.00 (56.00, 77.00) | 74.00 (63.00, 83.00) | <0.001 |
| Gender, n (%) |  |  |  | 0.038 |
| Female | 619 (43.90) | 336 (41.53) | 283 (47.09) |  |
| Male | 791 (56.10) | 473 (58.47) | 318 (52.91) |  |
| Race, n (%) |  |  |  | 0.021 |
| Non-White | 584 (41.42) | 314 (38.81) | 270 (44.93) |  |
| White | 826 (58.58) | 495 (61.19) | 331 (55.07) |  |
| **Vital signs** |  |  |  |  |
| Heart rate (beats/min) | 82 (71, 94) | 82 (71, 93) | 83 (71, 95) | 0.199 |
| SBP (mmHg) | 137 (122, 151) | 137 (124, 150) | 135 (119, 152) | 0.185 |
| DBP (mmHg) | 76 (65, 88) | 77 (67, 89) | 73 (63, 86) | 0.002 |
| Respiratory rate (times/min) | 18 (16, 22) | 18 (15, 21) | 19 (16, 22) | 0.011 |
| Temperature (℃) | 36.8 (36.5, 37.1) | 36.8 (36.6, 37.1) | 36.8 (36.5, 37.1) | 0.038 |
| SpO2 (%) | 98 (96, 100) | 98(96, 100) | 99 (96, 100) | <0.001 |
| **Severity scores** |  |  |  |  |
| SOFA | 3 (2, 5) | 2 (1, 4) | 4(2, 6) | <0.001 |
| GCS | 14 (11, 15) | 14 (12, 15) | 14 (10, 15) | 0.397 |
| LODS | 3 (2, 5) | 3 (2, 4) | 5 (3, 6) | <0.001 |
| **Comorbidity,n(%)** |  |  |  |  |
| Hypertension | 1135 (80.50) | 661 (81.71) | 474 (78.87) | 0.184 |
| Diabetes | 372 (26.38) | 205 (25.34) | 167 (27.79) | 0.302 |
| Myocardial infarction | 147 (10.43) | 77 (9.52) | 70 (11.65) | 0.196 |
| Congestive heart failure | 229 (16.24) | 114 (14.09) | 115 (19.13) | 0.011 |
| Peripheral vascular disease | 77 (5.46) | 42 (5.19) | 35 (5.82) | 0.605 |
| Chronic pulmonary disease | 176 (12.48) | 90 (11.12) | 86 (14.31) | 0.074 |
| AKI | 1083 (76.81) | 596 (73.67) | 487 (81.03) | 0.001 |
| Sepsis | 614 (43.55) | 283 (34.98) | 331 (55.07) | <0.001 |
| **Laboratory parameters** |  |  |  |  |
| WBC (K/μL) | 10.1 (7.8, 13.5) | 9.8 (7.5, 12.8) | 10.5 (8.2, 14.4) | <0.001 |
| Platelet (K/μL) | 200.0(155.0, 256.0) | 208.0 (168.0, 257.0) | 188.0 (141.0, 254.0) | <0.001 |
| BUN (mg/dL) | 17.0 (12.0, 23.0) | 15.0 (12.0, 21.0) | 19.0 (14.0, 26.0) | <0.001 |
| Creatinine (mg/dL) | 0.90 (0.70, 1.20) | 0.90 (0.70, 1.10) | 1.00 (0.70, 1.30) | 0.022 |
| Potassium (mmol/L) | 4.00 (3.60, 4.30) | 3.90 (3.60, 4.30) | 4.00 (3.70, 4.40) | 0.001 |
| Sodium (mmol/L) | 139.0 (137.0, 142.0) | 140.0 (137.0, 142.0) | 139.0 (136.0, 142.0) | 0.101 |
| Glucose (g/dL) | 130.0 (107.0, 161.0) | 124.0 (104.0, 149.0) | 139.0 (114.0, 177.0) | <.001 |
| INR | 1.20 (1.10, 1.30) | 1.10 (1.10, 1.20) | 1.20 (1.10, 1.40) | <.001 |
| Anion gap (mmol/L) | 15.00 (13.00, 17.00) | 14.00 (12.00, 16.00) | 15.00 (13.00, 17.00) | 0.004 |
| PT(s) | 12.70 (11.70, 14.40) | 12.40 (11.60, 13.80) | 13.30 (12.00, 15.10) | <.001 |
| **Treatment, n(%)** |  |  |  |  |
| Beta blockers | 580 (41.13) | 332 (41.04) | 248 (41.26) | 0.932 |
| Diuretic | 486 (34.47) | 253 (31.27) | 233 (38.77) | 0.003 |
| Mannitol | 187 (13.26) | 69 (8.53) | 118 (19.63) | <0.001 |
| Statin | 446 (31.63) | 276 (34.12) | 170 (28.29) | 0.020 |
| Vasoactive drug | 263 (18.65) | 90 (11.12) | 173 (28.79) | <0.001 |
| Ventilator | 980 (69.50) | 523 (64.65) | 457 (76.04) | <0.001 |
| CRRT | 46 (3.26) | 8 (0.99) | 38 (6.32) | <0.001 |
| Cerebral Surgery | 158 (11.21) | 76 (9.39) | 82 (13.64) | 0.012 |
| **Outcomes** |  |  |  |  |
| Hospital stay (day) | 8.57 (4.18, 16.13) | 9.01 (4.89, 17.81) | 7.44 (3.39, 14.92) | <0.001 |
| ICU stay (day) | 3.92 (1.88, 8.62) | 3.85 (1.86, 8.19) | 4.06 (1.93, 9.10) | 0.311 |

Abbreviations: RAR, red blood cell distribution width to albumin ratio; SBP, Systolic Blood Pressure; DBP, Diastolic Blood Pressure; SpO2, oxygen saturation;SOFA, sequential organ failure assessment; GCS, Glasgow coma scale; LODS, Logistic Organ Dysfunction System; AKI, acute kidney injury; WBC, white blood cell; BUN, blood urea nitrogen; INR, international normalized ratio; PT, prothrombin time; CRRT, continuous renal replacement therapy; ICU, intensive care unit.

| **Table S3**. **Covariance analysis between variables.** | |
| --- | --- |
| **Variables** | **VIF** |
| Age | 1.229 |
| Race | 1.081 |
| Gender | 1.045 |
| SOFA | 2.69 |
| LODS | 2.27 |
| Sepsis | 1.428 |
| BUN | 1.306 |
| Potassium | 1.095 |
| Glucose | 1.148 |
| Vasoactive drugs | 1.541 |
| Statin | 1.092 |
| Mannitol | 1.17 |
| CRRT | 1.481 |
| Cerebral surgery | 1.197 |
| Length of hospital stay | 1.245 |
| Abbreviations: VIF: variance inflation factor; SOFA, sequential organ failure assessment; LODS, Logistic Organ Dysfunction System; BUN, blood urea nitrogen; CRRT, continuous renal replacement therapy. | |

Table S4. Proportional hazards assumption test results.

| **Variables** | **chisq** | **df** | **P-value** |
| --- | --- | --- | --- |
| RAR | 6.513 | 1 | 0.011 |
| Age | 5.969 | 1 | 0.015 |
| Race | 5.512 | 1 | 0.019 |
| Gender | 0.073 | 1 | 0.787 |
| SOFA | 0.18 | 1 | 0.672 |
| LODS | 0.009 | 1 | 0.924 |
| Sepsis | 34.737 | 1 | <0.001 |
| BUN | 5.855 | 1 | 0.016 |
| Potassium | 0.997 | 1 | 0.318 |
| Glucose | 8.546 | 1 | 0.003 |
| Vasoactive drugs | 1.161 | 1 | 0.281 |
| Statin | 23.807 | 1 | <0.001 |
| Mannitol | 8.007 | 1 | 0.005 |
| CRRT | 16.396 | 1 | <0.001 |
| Cerebral surgery | 6.416 | 1 | 0.011 |
| Length of hospital stay | 307.36 | 1 | <0.001 |
| GLOBAL | 403.71 | 16 | <0.001 |

Abbreviations: RAR, red blood cell distribution width to albumin ratio; SOFA, sequential organ failure assessment; LODS, Logistic Organ Dysfunction System; BUN, blood urea nitrogen; CRRT, continuous renal replacement therapy.

| Table S5 Comparative Performance of RDW, Albumin, and RAR in fully adjusted Models for 90-day and 365-day mortality | | | | | | | | |
| --- | --- | --- | --- | --- | --- | --- | --- | --- |
| **Model** | **AUC** | |  | **NRI** | |  | **IDI** | |
|  | **Index (95% CI)** | **P value for Δ AUC** |  | **Index (95% CI)** | **p-value** |  | **Index (95% CI)** | **p-value** |
| **90-day mortality** |  |  |  |  |  |  |  |  |
| Model 3 with RDW | 0.822 (0.799, 0.844) |  |  |  |  |  |  |  |
| Model 3 with RAR | 0.831 (0.809, 0.853) | 0.004 |  | 0.0355 (0.0061-0.0649) | 0.018 |  | 0.0117 (0.0048-0.0187) | <0.001 |
| Model 3 with albumin | 0.831 (0.809, 0.853) |  |  |  |  |  |  |  |
| Model 3 with RAR | 0.831 (0.809, 0.853) | 0.896 |  | -0.0245 (-0.0555-0.0065) | 0.121 |  | -0.0035(-0.0083-0.0012) | 0.147 |
| **365-day mortality** |  |  |  |  |  |  |  |  |
| Model 3 with RDW | 0.797 (0.744, 0.820) |  |  |  |  |  |  |  |
| Model 3 with RAR | 0.805 (0.782, 0.828) | 0.017 |  | 0.0367 (0.0054-0.0681) | 0.022 |  | 0.0116 (0.0054-0.0179) | <0.001 |
| Model 3 with albumin | 0.802 (0.779, 0.825) |  |  |  |  |  |  |  |
| Model 3 with RAR | 0.805 (0.782, 0.828) | 0.24 |  | 0.0148(-0.0128-0.0425) | 0.293 |  | 0.0004 (-0.0032-0.005) | 0.673 |

Model 3 include age, gender, race SOFA, LODS, sepsis, BUN, Potassium, Glucose, Vasoactive drug, Statin, Mannitol, CRRT，Cerebral surgery, Length of stay in hospital

Abbreviations: RDW, red blood cell distribution width; RAR, red blood cell distribution width to albumin ratio; AUC, area under the curve; NRI,net reclassification improvement; IDI, integrated discrimination improvement;SOFA, sequential organ failure assessment; LODS, Logistic Organ Dysfunction System; BUN, blood urea nitrogen; CRRT, continuous renal replacement therapy.

Table S6 The nonlinear relationship between RAR and all-cause mortality

| **Models** | **HR (95%CI)** | **P-value** |
| --- | --- | --- |
| **90-day mortality** |  |  |
| Infection point | 4.82 |  |
| Fitting model by two-piecewise linear regression |  |  |
| <4.82 | 1.41(1.14, 1.73) | 0.001 |
| ≥4.82 | 1.00 (0.92,1.08) | 0.913 |
| Likelihood Ratio test |  | <0.001 |
| **365-day mortality** |  |  |
| Infection point | 4.73 |  |
| Fitting model by two-piecewise linear regression |  |  |
| <4.73 | 1.37 (1.13,1.66) | 0.002 |
| ≥4.73 | 1.01(0.94,1.08) | 0.783 |
| Likelihood Ratio test |  | <0.001 |

| Table S7 Time-varying Cox regression model for 90-day and 365-day mortality using raw data. | | | | | | | | |
| --- | --- | --- | --- | --- | --- | --- | --- | --- |
| **Variable** | **Model 1** | |  | **Model 2** | |  | **Model 3** | |
|  | **HR (95%CI)** | **P-value** |  | **HR (95%CI)** | **P-value** |  | **HR (95%CI)** | **P-value** |
| **90-day mortality** |  |  |  |  |  |  |  |  |
| RAR (per unit) | 1.17 (1.07~1.28) | <0.001 |  | 1.17 (1.07~1.28) | 0.001 |  | 1.24 (1.11~1.38) | <0.001 |
| RAR (quartiles) |  |  |  |  |  |  |  |  |
| Q1(RAR<3.22) | 1(ref) |  |  | 1(ref) |  |  | 1(ref) |  |
| Q2(3.22≤RAR<3.66) | 1.39 (0.67~2.86) | 0.379 |  | 1.40 (0.68~2.89) | 0.366 |  | 1.61 (0.77~3.36) | 0.202 |
| Q3(3.66≤RAR<4.39) | 1.64 (0.83~3.25) | 0.157 |  | 1.64 (0.83~3.25) | 0.156 |  | 1.70 (0.85~3.39) | 0.133 |
| Q4(4.39≤RAR) | 2.20 (1.15~4.19) | 0.017 |  | 2.17 (1.14~4.15) | 0.019 |  | 2.61 (1.36~5.01) | 0.004 |
| *P* for trend |  | <0.001 |  |  | <0.001 |  |  | <0.001 |
| **365-day mortality** |  |  |  |  |  |  |  |  |
| RAR (per unit) | 1.13 (1.10~1.17) | <0.001 |  | 1.14 (1.10~1.18) | <0.001 |  | 1.12 (1.06~1.18) | <0.001 |
| RAR (quartiles) |  |  |  |  |  |  |  |  |
| Q1(RAR<3.22) | 1(ref) |  |  | 1(ref) |  |  | 1(ref) |  |
| Q2(3.22≤RAR<3.66) | 1.35 (0.98~1.87) | 0.069 |  | 1.27 (0.91~1.75) | 0.156 |  | 1.12 (0.80~1.55) | 0.51 |
| Q3(3.66≤RAR<4.39) | 1.87 (1.38~2.54) | <0.001 |  | 1.69 (1.25~2.30) | 0.001 |  | 1.31 (0.96~1.80) | 0.09 |
| Q4(4.39≤RAR) | 2.89 (2.16~3.86) | <0.001 |  | 2.72 (2.04~3.64) | <0.001 |  | 1.73 (1.26~2.37) | 0.001 |
| *P* for trend |  | <0.001 |  |  | <0.001 |  |  | <0.001 |
| Model 1: unadjusted.  Model 2: adjusted for age, gender, race  Model 3:adjusted for Model 2 plus SOFA, LODS, sepsis, BUN, Potassium, Glucose, Vasoactive drug, Statin, Mannitol, CRRT，Cerebral surgery, Length of stay in hospital  Abbreviations: RAR, red blood cell distribution width to albumin ratio; SOFA, sequential organ failure assessment; LODS, Logistic Organ Dysfunction System; BUN, blood urea nitrogen; CRRT, continuous renal replacement therapy. | | | | | | | | |


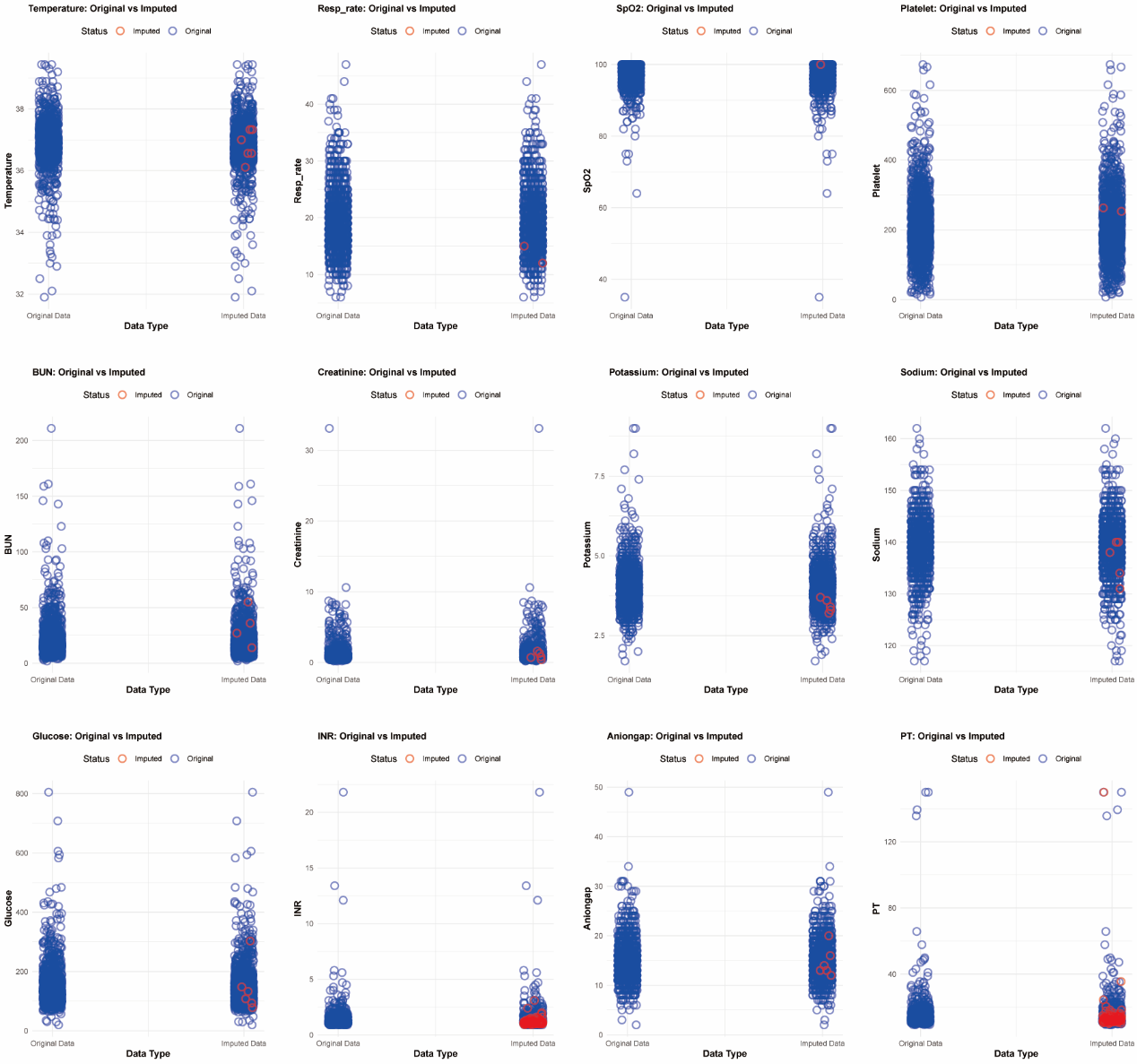


Figure S1 Comparison between the original and imputed data. The non-missing values of the original data are shown in blue, while the imputed data are shown in red. The high degree of overlap between the imputed and observed data indicates good imputation quality.

| 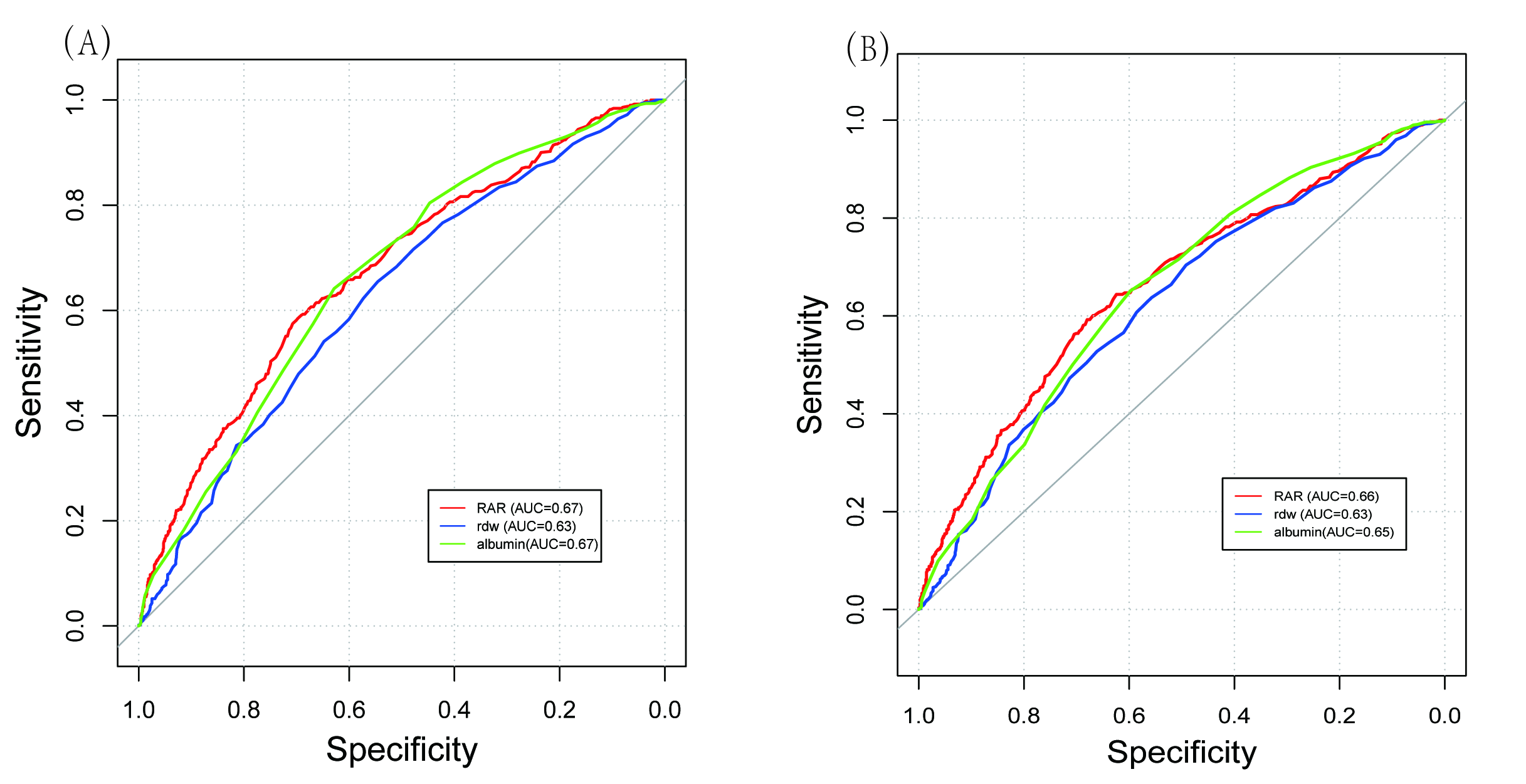 |
| --- |
| Figure S2 Receiver operating characteristic (ROC) curves for predicting all-cause mortality. (a)90-day all-cause mortality. (b)365-day mortality. RAR, red blood cell distribution width to albumin ratio; RDW, red cell distribution width. |

| 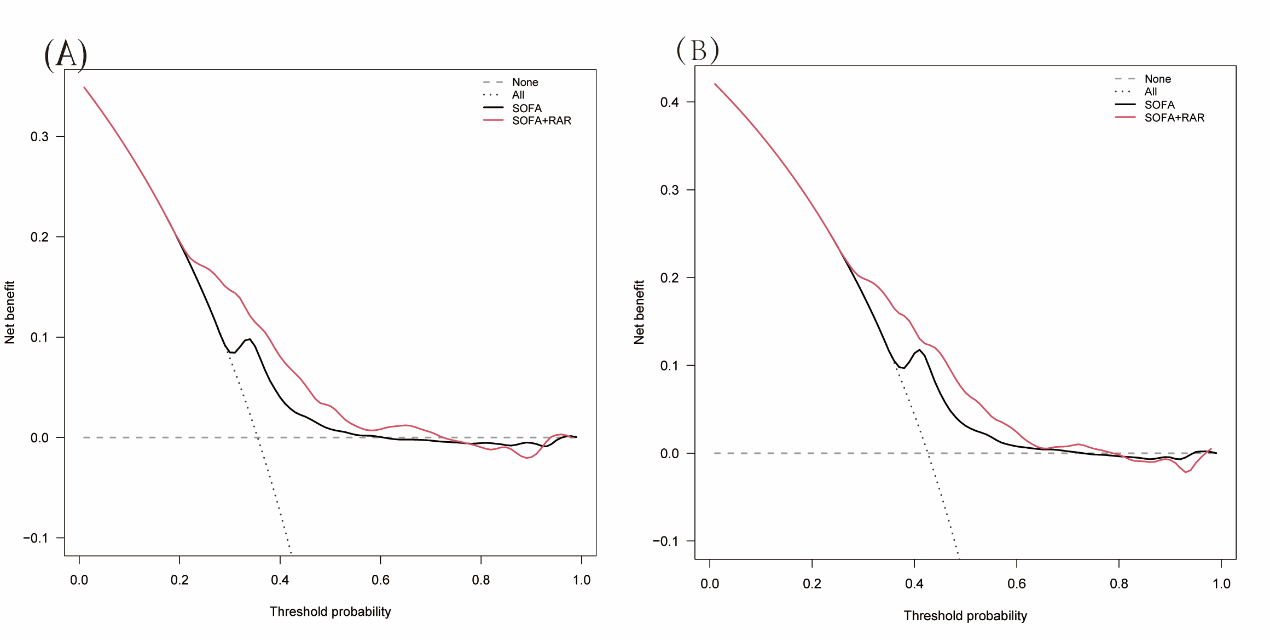 |
| --- |
| Figure S3. Decision curve analysis (DCA) for 90-day(A) and 365-day mortality(B) prediction. The SOFA and RAR model shows improved net clinical benefit relative to the SOFA model over a wide threshold range. SOFA, sequential organ failure assessment; RAR, red blood cell distribution width to albumin ratio. |
